# Enhanced Recovery After Surgery (ERAS®) Protocol in Colorectal Resections: A Prospective Observational Study of Implementation and Outcomes at a Tertiary Referral Center

**DOI:** 10.7759/cureus.88664

**Published:** 2025-07-24

**Authors:** Nadeem Ahmad, Kanchan Sone Lal Baitha, Shashi S Pawar, Farhan Mohsin, Prem Prakash, Rishika Raj, Saptarshi Mondal

**Affiliations:** 1 General Surgery, Indira Gandhi Institute of Medical Sciences (IGIMS) Patna, Patna, IND; 2 Surgical Oncology, Indira Gandhi Institute of Medical Sciences (IGIMS) Patna, Patna, IND

**Keywords:** clavien-dindo classification, colorectal resection, enhanced recovery after surgery, eras compliance, postoperative complications

## Abstract

Aim: This study aimed to evaluate the implementation of the Enhanced Recovery After Surgery (ERAS®) protocol in patients undergoing colorectal cancer resections at a tertiary care center in India, and to assess whether protocol compliance influences the length of hospital stay and postoperative complications.

Materials and methods: We conducted a prospective observational study of 50 consecutive patients undergoing colorectal cancer resection surgery at Indira Gandhi Institute of Medical Sciences (IGIMS), Patna, between May 2024 and April 2025. A 16-item ERAS® protocol was implemented, and compliance was measured. Primary outcomes included the length of hospital stay, postoperative complications (classified according to the Clavien-Dindo classification), and 30-day readmission rates.

Results: Overall compliance with the ERAS® protocol was 78.6% across all 50 patients. Mean length of stay was 6.3 ± 2.7 days, with significantly shorter stays in patients who achieved >80% compliance (24/50 patients, 5.2 ± 1.8 days) compared with those below this threshold (26/50, 7.6 ± 3.0 days; p=0.003). The overall complication rate was 24% (12/50), while major complications (Clavien-Dindo grade ≥ III) occurred in 8% (4/50). The 30-day readmission rate was 4% (2/50). Greater compliance was associated with fewer overall complications (4/24 (16.7%) vs. 11/26 (42.3%); p=0.038), although it did not significantly influence major complications or readmissions.

Conclusions: The implementation of the ERAS® protocol in colorectal cancer surgery is feasible in a resource-constrained setting, with acceptable compliance rates. Higher compliance was associated with shorter hospital stay and reduced overall complication rates. The protocol can be safely applied to patients undergoing colorectal cancer surgeries, potentially improving outcomes and resource utilization.

## Introduction

Enhanced Recovery After Surgery (ERAS®) programs, introduced in the 1990s, have transformed perioperative care for patients undergoing major surgical procedures. The concept underlying ERAS® is to apply evidence-based perioperative practices to improve postoperative recovery, reduce surgical stress, maintain physiological function, and ultimately shorten hospital stay without increasing complications or readmissions [1,2]. Colorectal surgery encompasses complex procedures that have historically been surrounded by dogmas and practices lacking scientific evidence. Traditional perioperative practices often included prolonged fasting, mechanical bowel preparation, routine nasogastric tube placement, delayed oral feeding, and extended bed rest [3-5]. These practices have been questioned and systematically evaluated through randomized controlled trials and meta-analyses, resulting in evidence-based ERAS® protocols.

The creation of the term ERAS® and its conceptual development emerged in 2001 when a group of European surgeons met in London to develop evidence-based perioperative management guidelines [6]. Kehlet and Mogensen had previously published groundbreaking work demonstrating the possibility of early hospital discharge in patients undergoing sigmoid colon resections, a radical departure from standard practice at that time [7]. After the formation of the ERAS® Society and the publication of the first recommendations for colonic surgery by Gustafsson et al. in 2012, the concept gained international recognition and adoption [8]. The International Surgical Outcomes Study reported that globally, 26.8% of patients undergoing significant surgery experience postoperative complications, with 24.3% of those undergoing lower abdominal surgery developing complications [9]. Postoperative complications following colorectal surgery have been associated with decreased long-term survival, independent of patient, disease, and treatment factors [10].

Several studies from developed countries have demonstrated significant benefits of ERAS® protocols in colorectal surgery, including reduced length of stay, decreased complications, faster return of gastrointestinal function, and cost savings [11-13]. Ota et al. demonstrated earlier oral diet resumption, faster return of gastrointestinal function, and earlier discharge in patients managed with ERAS® protocols in Japan [14]. Greco et al. demonstrated that ERAS® pathways reduced overall morbidity rates and shortened hospital stays without increasing readmission rates, particularly by reducing non-surgical complications [15]. Thiele et al. achieved significant reductions in hospital stay, complication rates, and costs through the implementation of ERAS® [16].

The safety and applicability of ERAS® protocols in specific patient populations, such as the elderly and those with poor performance status, have also been addressed. Koh et al. [17] found ERAS® protocols safe and feasible in colorectal cancer patients aged over 70 years, while Braga et al. [18] confirmed that ERAS® pathways could be safely applied in elderly and low physical status patients, with only slight differences in outcomes. Despite growing evidence supporting ERAS® protocols in colorectal surgery worldwide, implementation data from resource-limited settings remains sparse. Furthermore, understanding factors that influence protocol compliance and the relationship between compliance and outcomes remains critical for successful implementation.

This prospective observational study aims to evaluate the implementation of a 16-item ERAS® protocol in patients undergoing colorectal cancer resections at a tertiary care center in India. Specifically, we aim to determine overall compliance rates, identify factors that influence compliance, and assess whether compliance affects important clinical outcomes, including length of hospital stay, postoperative complications, and readmission rates. Such data will be valuable for guiding ERAS® implementation efforts in similar resource-constrained settings.

## Materials and methods

Study design and setting

This prospective observational study was conducted at the Department of General Surgery and the Department of Surgical Oncology at the Indira Gandhi Institute of Medical Sciences (IGIMS), Patna, Bihar, India, from May 2024 to April 2025.

Inclusion and exclusion criteria

All adult patients (≥18 years) who were scheduled for an elective colorectal resection for malignant pathology and who could understand and freely consent to follow the ERAS® pathway were eligible for inclusion. Patients were excluded if they presented with distant metastases that necessitated multi-organ resection, required emergency surgery (e.g., obstruction or perforation), were receiving preoperative immunosuppression (e.g., chemotherapy administered within four weeks of the scheduled surgery or systemic steroids), or had an American Society of Anesthesiologists (ASA) physical status classification of IV or V.

Methodology

ERAS® Protocol Implementation

A standardized 16-item ERAS® protocol was implemented for all patients included in the study. This protocol was adapted from ERAS® Society guidelines and tailored to local resources and practices [8]. The protocol components are detailed in Table 1. Before implementation, a multidisciplinary team consisting of surgeons, anesthesiologists, nurses, nutritionists, and physiotherapists was formed. Team members underwent training on ERAS® principles and protocol components. Educational materials were developed for patients and caregivers. For each patient, compliance with individual protocol elements was documented using a standardized checklist. Overall compliance was calculated as the percentage of protocol elements successfully implemented for each patient.

**Table 1:** Components of the ERAS® protocol implemented in the study TME: total mesorectal excision, POD: postoperative day, TAP: transversus abdominis plane, NSAIDs: nonsteroidal anti-inflammatory drugs, IV: intravenous, TME: total mesorectal excision, TAP: transversus abdominis plane, NSAIDs: nonsteroidal anti-inflammatory drugs Gustafsson et al., 2019 [8]

No.	Protocol element	Description
1	Preoperative counseling and patient education	Detailed information about diagnosis, procedure, and recovery expectations
2	No bowel preparation	No routine mechanical bowel preparation except in cases of low rectal resection involving TME and creation of a defunctioning loop ileostomy
3	Preoperative carbohydrate loading	Clear carbohydrate drink (400 ml, oral) evening before surgery and 2-3 hours before induction
4	Antithrombotic prophylaxis	Enoxaparin 40 mg subcutaneously starting the evening before surgery
5	Antibiotic prophylaxis	Administered 30-60 minutes prior to skin incision
6	Balanced intravenous fluid therapy	<2500ml intravenous fluids during surgery, <150 mmol sodium
7	No nasogastric tubes postoperatively	Removal of the nasogastric tube at the end of surgery
8	No routine drains	Drains are placed only when necessary and removed within 24 hours when possible
9	TAP block	Performed at the end of surgery
10	Avoiding opioids, multimodal analgesia	Regular acetaminophen and NSAIDs, opioids only as rescue
11	Prevention of postoperative nausea and vomiting	Ondansetron 8 mg IV, metoclopramide 10 mg IV
12	Postoperative oxygenation therapy	Supplemental oxygen (4-6 L/min) for 24 hours
13	Early oral feeding	Oral supplements 4 hours postoperatively, light diet on POD 1, full diet on POD 2
14	Urinary catheter removal	Removal on postoperative day 1
15	Full mobilization on POD 1	Out of bed for a total of at least 4 hours (which may be cumulative), walking along a corridor
16	Discharge criteria	Tolerating solid food, passing flatus/stool, adequate pain control with oral analgesia, independently mobile, willing to go home

Perioperative Management

All surgeries were performed by surgeons experienced in colorectal procedures. The decision between open and laparoscopic approaches was made at the surgeon's discretion based on patient and tumor characteristics. Preoperative patient education was provided by the surgical team, which explained the diagnosis, the planned procedure, the expected recovery trajectory, and the patient's role in the recovery process. Nutritional status was assessed preoperatively using the Subjective Global Assessment tool, and nutritional supplementation was provided when necessary.

Patients received preoperative carbohydrate loading (400 ml of clear carbohydrate drink) the evening before surgery and two to three hours before induction of anesthesia. Mechanical bowel preparation was avoided except for patients undergoing low rectal resection with total mesorectal excision and defunctioning loop ileostomy. Thromboprophylaxis was initiated the evening before surgery with subcutaneous low molecular weight heparin (enoxaparin 40 mg) and continued throughout the hospital stay. Antibiotic prophylaxis was administered 30-60 minutes before skin incision. Intraoperative fluid management focused on a balanced approach, limiting crystalloid infusion to <2500 ml during surgery. Nasogastric tubes were removed immediately after surgery, and drains were avoided when possible or removed within 24 hours.

Multimodal analgesia, including transversus abdominis plane block, was utilized to minimize opioid use. Postoperative nausea and vomiting were managed prophylactically. Early oral feeding was initiated within four hours postoperatively with clear liquids and oral nutritional supplements, advancing to a light hospital diet on postoperative day 1 and a full diet on day 2. Urinary catheters were removed on postoperative day 1, and patients were out of bed for a total of at least four hours (which may be cumulative), walking along a corridor.

Clinical data collection

This study collected comprehensive data for each patient, beginning with baseline characteristics such as age, gender, BMI, comorbidities, ASA physical status, tumor location, and clinical staging. Operative details were meticulously recorded, including the surgical approach (open vs. laparoscopic), type of resection, total operative time, estimated blood loss, and any intraoperative complications that occurred. Following the procedure, a range of postoperative outcomes was assessed to gauge recovery, including the length of hospital stay, time to the return of bowel function, resumption of a solid diet, mobilization milestones, and pain levels measured using the Visual Analog Scale. Any postoperative complications were classified according to the Clavien-Dindo system, and readmissions within 30 days of discharge were also tracked. All patients were monitored for a 30-day period post-discharge through outpatient visits or telephone calls to ensure a complete data set.

Statistical analysis

The sample size was calculated based on previous studies indicating a mean length of stay of 8 ± 3 days with traditional care. An expected reduction to a mean of 6 ± 2 days with the implementation of the ERAS® protocol was informed by compliance-stratified outcomes in observational data, as reported by the ERAS® Compliance Group in 2015. With an alpha level set at 0.05 and a power of 80%, a minimum of 44 patients was required. To account for potential dropouts, 50 patients were planned for enrollment.

Data were analyzed using SPSS Statistics version 25.0 (IBM Corp. Released 2017. IBM SPSS Statistics for Windows, Version 25.0. Armonk, NY: IBM Corp.). Continuous variables were expressed as mean ± standard deviation or median with interquartile range, depending on data distribution. Categorical variables were presented as frequencies and percentages.

For the analysis of protocol compliance, patients were categorized into two groups: a high-compliance group (achieving ≥80% of protocol elements) and a moderate-compliance group (achieving <80% of protocol elements). The 80% compliance threshold was chosen based on established ERAS® literature, which indicates that adherence of ≥80% is associated with optimal outcomes, a finding supported by Pisarska et al. in 2016 and the ERAS® Compliance Group in 2015. Outcomes were compared between these groups using the Student's t-test or Mann-Whitney U test for continuous variables and the chi-square or Fisher's exact test for categorical variables. A multivariate logistic regression was performed to identify factors independently associated with postoperative complications. A p-value of <0.05 was considered statistically significant.

Ethical considerations

The Institutional Ethics Committee of Indira Gandhi Institute of Medical Sciences (IGIMS) approved the study protocol, Patna (approval number: 1200/IEC/IGIMS/2023, approval date: October 5, 2023), and written informed consent was obtained from all participants.

## Results

Demographic and clinical data

A total of 58 patients with colorectal cancer were assessed for eligibility during the study period. After exclusions (five patients with distant metastases, two requiring emergency surgery, and one requiring multi-organ resection), 50 patients were enrolled and completed the study. The baseline characteristics of the study population are presented in Table 2. The mean age was 58.4 ± 12.7 years (range: 31-82 years), with 64% of participants being male. Most patients (72%) had an ASA score of II. Comorbidities were present in 64% of patients, with hypertension (36%) and diabetes mellitus (24%) being the most common. The rectum (42%) was the most common location of tumors, followed by the sigmoid colon (22%), the ascending colon (16%), the descending colon (12%), and the transverse colon (8%). Clinical staging revealed 6% of patients had stage I disease, 42% had stage II, and 52% had stage III disease. Preoperative neoadjuvant chemoradiotherapy had been administered to 18 patients (36%), all with rectal cancer. Neoadjuvant therapy was completed at least four weeks prior to surgery in all cases, as per the exclusion criteria. Although the initial protocol allowed for benign pathologies, all enrolled patients during the study period had malignant disease, reflecting the predominant case mix at our institution.

**Table 2:** Baseline characteristics of the study population (n=50) p-values calculated using an independent t-test for continuous variables and a chi-square test (or Fisher's exact test for expected counts <5) for categorical variables. BMI: body mass index, ASA: American Society of Anesthesiologists, COPD: chronic obstructive pulmonary disease, SD: standard deviation, g/dL: grams per deciliter

Characteristic	Value n(%)
Age (years)	
- Mean ± SD	58.4 ± 12.7
- Range	31–82
- Age >70 years, n (%)	12 (24)
Gender, n (%)	
- Male	32 (64)
- Female	18 (36)
BMI (kg/m²)	
- Mean ± SD	23.6 ± 3.8
- <18.5, n (%)	5 (10)
- 18.5–24.9, n (%)	29 (58)
- 25–29.9, n (%)	13 (26)
- ≥30, n (%)	3 (6)
ASA score, n (%)	
- I	8 (16)
- II	36 (72)
- III	6 (12)
Comorbidities, n (%)	
- Hypertension	18 (36)
- Diabetes mellitus	12 (24)
- Coronary artery disease	7 (14)
- COPD/Asthma	5 (10)
- Others	8 (16)
Tumor location, n (%)	
- Rectum	21 (42)
- Sigmoid colon	11 (22)
- Ascending colon	8 (16)
- Descending colon	6 (12)
- Transverse colon	4 (8)
Clinical stage, n (%)	
- Stage I	3 (6)
- Stage II	21 (42)
- Stage III	26 (52)
Neoadjuvant therapy, n (%)	18 (36)
Hemoglobin (g/dL), mean ± SD	11.2 ± 1.9
Albumin (g/dL), mean ± SD	3.6 ± 0.6

Operative parameters

Table 3 summarizes the operative parameters. Open surgery was performed on 36 patients (72%), while 14 patients (28%) underwent laparoscopic procedures. While the open and laparoscopic approaches were not randomized, the surgical approach was controlled for as a potential confounder in the multivariate analysis of outcomes. The most common procedure was anterior resection (36%), followed by right hemicolectomy (26%), sigmoid colectomy (14%), abdominoperineal resection (12%), left hemicolectomy (8%), and total colectomy (4%). Procedures such as abdominoperineal resection, which involve both abdominal and perineal incisions, were compared descriptively but not as primary endpoints due to these inherent differences.

**Table 3:** Operative parameters (n=50) p-values calculated using an independent t-test for continuous variables and a chi-square test (or Fisher's exact test for expected counts <5) for categorical variables. SD: standard deviation, ml: milliliters, min: minutes

Parameter	Overall (n=50)	High compliance (n=24)	Moderate compliance (n=26)	p-value
Surgical approach, n (%)				
Open	36 (72)	16 (66.7)	20 (76.9)	0.416
Laparoscopic	14 (28)	8 (33.3)	6 (23.1)	-
Type of resection, n (%)				
Anterior resection	18 (36)	9 (37.5)	9 (34.6)	0.825
Right hemicolectomy	13 (26)	6 (25.0)	7 (26.9)	0.876
Sigmoid colectomy	7 (14)	3 (12.5)	4 (15.4)	0.764
Abdominoperineal resection	6 (12)	3 (12.5)	3 (11.5)	0.914
Left hemicolectomy	4 (8)	2 (8.3)	2 (7.7)	0.937
Total colectomy	2 (4)	1 (4.2)	1 (3.8)	0.949
Stoma creation, n (%)				
Loop ileostomy	10 (20)	5 (20.8)	5 (19.2)	0.886
End colostomy	4 (8)	2 (8.3)	2 (7.7)	0.937
Operative time (minutes)				
Mean ± SD	182 ± 45	170 ± 40	193 ± 47	0.05
Open approach	165 ± 37	158 ± 34	171 ± 39	0.12
Laparoscopic approach	226 ± 31	220 ± 28	234 ± 34	0.198
Operative time >240 min, n (%)	10 (20)	2 (8.3)	8 (30.8)	0.028
Estimated blood loss (ml)				
Mean ± SD	310 ± 170	280 ± 150	338 ± 183	0.152
Open approach	350 ± 180	320 ± 162	377 ± 192	0.18
Laparoscopic approach	210 ± 90	195 ± 81	230 ± 99	0.215
Intraoperative complications, n (%)	3 (6)	1 (4.2)	2 (7.7)	0.593
Bleeding requiring transfusion	2 (4)	1 (4.2)	1 (3.8)	0.949
Splenic injury	1 (2)	0 (0)	1 (3.8)	0.333

A protective stoma was created in 14 patients (28%), including 10 with loop ileostomy and four with end colostomy. Intraoperative complications occurred in three patients (6%), including bleeding requiring transfusion (two patients) and inadvertent splenic injury (one patient).

ERAS® protocol compliance

Overall compliance with the 16-item ERAS® protocol was 78.6% across all patients. Compliance with individual protocol elements is detailed in Table 4. The highest compliance (>90%) was observed for preoperative counseling, antibiotic prophylaxis, avoidance of nasogastric tubes, and prevention of postoperative nausea and vomiting. The lowest compliance (<70%) was noted for transversus abdominis plane block, early removal of urinary catheters, and full mobilization on postoperative day 1. Twenty-four patients (48%) achieved high compliance (≥80% of protocol elements), while 26 patients (52%) had moderate compliance (<80% of protocol elements).

**Table 4:** Compliance with individual ERAS® protocol elements (n=50) * Excluding patients with planned stoma (n=14) PONV: postoperative nausea and vomiting, POD: postoperative day, IV: intravenous, TAP: transversus abdominis plane, ERAS®: Enhanced Recovery After Surgery Gustafsson et al., 2019 [8]

Protocol element	Number of patients (%)
Preoperative counseling	48 (96)
No bowel preparation*	42 (84)
Preoperative carbohydrate loading	39 (78)
Antithrombotic prophylaxis	46 (92)
Antibiotic prophylaxis	50 (100)
Balanced IV fluid therapy	41 (82)
No nasogastric tubes postoperatively	47 (94)
No drains/early removal	38 (76)
TAP block	31 (62)
Multimodal analgesia, limiting opioids	43 (86)
Prevention of PONV	49 (98)
Postoperative oxygenation therapy	47 (94)
Early oral feeding	40 (80)
Early urinary catheter removal	33 (66)
Full mobilization on POD 1	32 (64)
Overall compliance rate	78.6%

Patient factors associated with lower compliance included age >70 years (p=0.021), ASA III status (p=0.034), presence of multiple comorbidities (p=0.042), and extended operative time >240 minutes (p=0.028).

Primary outcomes

Length of Hospital Stay

The mean postoperative length of stay (LOS) was 6.3 ± 2.7 days (range: 3-14 days), with a median of 5 days. Patients in the high compliance group had a significantly shorter mean LOS compared to those in the moderate compliance group (5.2 ± 1.8 vs. 7.6 ± 3.0 days, p=0.003). Multivariate analysis identified factors independently associated with prolonged LOS (>7 days), including age >70 years (OR 2.67, 95% CI 1.24-5.73, p=0.012), ASA III status (OR 2.18, 95% CI 1.06-4.48, p=0.034), presence of postoperative complications (OR 4.83, 95% CI 2.15-10.86, p<0.001), and protocol compliance <80% (OR 3.21, 95% CI 1.46-7.05, p=0.004).

Subgroup analysis by surgical approach showed no significant difference in mean LOS (open: 6.5 ± 2.9 days vs. laparoscopic: 5.8 ± 2.1 days, p=0.312) or overall complication rates (open: 25% (9/36) vs. laparoscopic: 21% (3/14), p=0.745). Surgical approach was included as a covariate in multivariate models to control for potential confounding.

Postoperative Complications

Postoperative complications occurred in 12 patients (24%), with details provided in Table 5. Using the Clavien-Dindo classification, eight patients (16%) had grade I-II complications, and four patients (8%) experienced grade III-V complications.

**Table 5:** Postoperative complications according to the Clavien-Dindo classification (n=50) Grade I-II: minor complications not requiring surgical, endoscopic, or radiological intervention. Grade III-V: major complications requiring intervention (grade III), life-threatening (grade IV), or resulting in death (grade V).

Complication	n (%)	Clavien-Dindo grade
Grade I-II	8 (16)	
Ileus	3 (6)	I
Surgical site infection	3 (6)	I-II
Urinary tract infection	2 (4)	II
Grade III-V	4 (8)	
Anastomotic leak requiring reoperation	2 (4)	IIIb
Pneumonia with respiratory failure	1 (2)	IVa
Myocardial infarction (fatal)	1 (2)	V
Total complications	12 (24)	

The most common complications were ileus (6%), surgical site infection (6%), and urinary tract infection (4%). Major complications included anastomotic leak requiring reoperation (4%), pneumonia with respiratory failure (2%), and myocardial infarction (2%). The complication rate was significantly lower in the high-compliance group compared to the moderate-compliance group (16.7% vs. 31.8%, p=0.038). However, when analyzing only major complications (Clavien-Dindo grade ≥III), the difference was not statistically significant (5.3% vs. 10.7%, p=0.273).

Multivariate analysis identified independent risk factors for postoperative complications: age >70 years (OR 2.45, 95% CI 1.18-5.09, p=0.017), ASA III status (OR 2.03, 95% CI 1.02-4.05, p=0.044), operative time >240 minutes (OR 2.67, 95% CI 1.28-5.57, p=0.009), and protocol compliance <80% (OR 2.26, 95% CI 1.12-4.56, p=0.023) (Tables 6-7).

**Table 6:** Multivariate analysis of factors associated with prolonged LOS (>7 days) ASA: American Society of Anesthesiologists, ERAS®: Enhanced Recovery After Surgery, OR: odds ratio, CI: confidence interval, LOS: length of stay

Variable	OR	95% CI	p-value
Age >70 years	2.67	1.24-5.73	0.012
ASA III status	2.18	1.06-4.48	0.034
Any postoperative complication	4.83	2.15-10.86	<0.001
ERAS® compliance <80%	3.21	1.46-7.05	0.004
Surgical approach (open vs. laparoscopic)	1.15	0.52-2.54	0.728

**Table 7:** Multivariate analysis of factors associated with any postoperative complication ASA: American Society of Anesthesiologists, ERAS®: Enhanced Recovery After Surgery, OR: odds ratio, CI: confidence interval, LOS: length of stay

Variable	OR	95% CI	p-value
Age >70 years	2.45	1.18-5.09	0.017
ASA III status	2.03	1.02-4.05	0.044
Operative time >240 min	2.67	1.28-5.57	0.009
ERAS® compliance <80%	2.26	1.12-4.56	0.023
Surgical approach (open vs. laparoscopic)	1.12	0.48-2.61	0.792

Readmission Rate

Two patients (4%) required readmission within 30 days of discharge. One patient was readmitted for anastomotic stricture requiring endoscopic dilation, and the other for intra-abdominal collection managed with percutaneous drainage. There was no significant difference in readmission rates between high and moderate compliance groups (2.4% vs. 5.7%, p=0.412).

Secondary outcomes

Recovery milestones are summarized in Table 8. The mean time to first flatus was 1.8 ± 0.9 days, first bowel movement 2.9 ± 1.2 days, and tolerance of solid diet 2.4 ± 1.1 days. Patients in the high-compliance group demonstrated shorter times to first flatus (1.5 vs. 2.1 days, p=0.032), earlier bowel movements (2.5 vs. 3.3 days, p=0.027), and earlier tolerance of a solid diet (2.0 vs. 2.8 days, p=0.018) compared to the moderate-compliance group. Pain scores (measured on a Visual Analog Scale of 0-10) were significantly lower in the high compliance group on postoperative days 1 and 2 (4.2 vs. 5.7, p=0.014; and 2.8 vs. 3.9, p=0.023, respectively). Mobilization milestones were achieved earlier in the high-compliance group, with more patients able to sit out of bed on postoperative day 1 (95.8% vs. 76.9%, p=0.041) and ambulate on postoperative day 2 (87.5% vs. 65.4%, p=0.049).

**Table 8:** Recovery milestones based on protocol compliance * Statistically significant (p<0.05) VAS: Visual Analog Scale, POD: postoperative day

Parameter	Overall (n=50)	High compliance (n=24)	Moderate compliance (n=26)	Test statistic (t / χ²)	p-value
Time to first flatus (days)	1.8 ± 0.9	1.5 ± 0.7	2.1 ± 1.0	t = -2.47	0.032*
Time to first bowel movement (days)	2.9 ± 1.2	2.5 ± 0.9	3.3 ± 1.3	t = -2.55	0.027*
Time to solid diet tolerance (days)	2.4 ± 1.1	2.0 ± 0.8	2.8 ± 1.2	t = -2.79	0.018*
Pain score (VAS) POD 1	5.0 ± 1.8	4.2 ± 1.6	5.7 ± 1.7	t = -3.21	0.014*
Pain score (VAS) POD 2	3.4 ± 1.5	2.8 ± 1.3	3.9 ± 1.4	t = -2.88	0.023*
Out of bed on POD 1, n (%)	43 (86)	23 (95.8)	20 (76.9)	χ² = 3.71	0.041*
Ambulation on POD 2, n (%)	38 (76)	21 (87.5)	17 (65.4)	χ² = 3.35	0.049*
Length of stay (days)	6.3 ± 2.7	5.2 ± 1.8	7.6 ± 3.0	t = –3.46	0.003*
Readmission, n (%)	2 (4)	1 (2.4)	1 (5.7)	χ² = 0.32	0.412

Among the 50 patients, 30-day mortality occurred in one patient (2%) due to myocardial infarction on postoperative day 5. This patient was in the moderate compliance group.

## Discussion

Our study demonstrates the successful implementation of ERAS® protocols in colorectal cancer surgery within a resource-constrained setting, achieving an overall compliance rate of 78.6%, comparable to that of established centers in high-income countries [8,19]. Protocol elements showing the highest compliance (>90%) included preoperative counseling, antibiotic prophylaxis, and avoidance of nasogastric tubes. Challenges were encountered with TAP blocks, early catheter removal, and early mobilization (<70%).

Several large meta-analyses report mean LOS reductions of 2-3 days and absolute complication rate decreases of 8-12% following the adoption of ERAS® [11,15]. Our mean LOS (6.3 days) and 24% overall complication rate align closely with the figures described by Greco et al. and Zhuang et al., who noted pooled LOS values of 5.8-6.5 days and complication rates of approximately 23%. Notably, our high-compliance cohort achieved an LOS of 5.2 days, virtually identical to the 5.1 days reported by Thiele et al. in a high-resource U.S. setting, demonstrating that comparable efficiencies are attainable even in low-to-middle-income environments. Multivariate logistic regression further underscored the importance of protocol adherence: ERAS® compliance <80% independently predicted both prolonged LOS >7 days (OR 3.21; 95% CI 1.46-7.05) and occurrence of any postoperative complication (OR 2.26; 95% CI 1.12-4.56) (Tables 7-8). Conversely, patients who met ≥80% of ERAS® items experienced nearly threefold lower odds of extended hospitalization and twofold lower odds of complications.

Key barriers to full compliance included limited specialist availability, staff workload constraints, and cultural factors affecting patient participation. Higher compliance (≥80%) was significantly correlated with shorter hospital stays (5.2 vs. 7.6 days, p=0.003) and fewer complications (16.7% vs. 31.8%, p=0.038), consistent with findings from previous studies [20]. Our 4% readmission rate compares favorably with literature reports (3-8%) [12,15,21], with no significant difference between compliance groups. Patients with higher protocol compliance experienced faster gastrointestinal recovery, earlier tolerance to diet, better pain control, and improved mobilization. Patient-related factors associated with lower compliance included advanced age, higher ASA scores, multiple comorbidities, and extended operative time. Despite these challenges, even partial implementation benefited high-risk patients, supporting findings by Braga et al. [18] and Koh et al. [17].

Study limitations include its observational nature, relatively small sample size, single-center design, and lack of a control group. Nonetheless, the consistency of our results with large multi-center series suggests that context-adapted ERAS® pathways can yield meaningful improvements across diverse healthcare environments when multidisciplinary collaboration, continuous auditing, and feedback loops are maintained. A key limitation is potential selection bias, as moderate-compliance patients had higher risk profiles (e.g., older age, higher ASA score, multiple comorbidities, and longer operative times). While multivariate analysis adjusted for these factors, residual confounding may still exist, and the observed outcome differences may be partly attributable to these baseline factors rather than compliance alone.

## Conclusions

Implementation of an ERAS® protocol in colorectal cancer surgery is feasible in a resource-constrained setting, with acceptable compliance rates. Higher compliance was significantly associated with shorter hospital stays and reduced overall complication rates, without increasing readmission rates. The protocol can be safely applied to patients undergoing colorectal cancer surgeries, potentially improving outcomes and resource utilization. Our experience underscores the importance of multidisciplinary collaboration, context-specific protocol adaptation, and ongoing compliance monitoring to optimize the benefits of ERAS® implementation. Furthermore, larger multicenter studies are necessary to validate these findings and determine the optimal implementation strategies for various healthcare settings. The results of this study suggest that ERAS® protocols may be a valuable approach for patients undergoing colorectal cancer surgery, even in resource-constrained settings, and warrant further controlled trials. Future research should focus on identifying barriers to implementing specific protocol elements and developing targeted interventions to improve compliance, particularly in high-risk patient subgroups.
